# Video consultations: a potential gamechanger in clinical education

**DOI:** 10.3399/bjgp22X720509

**Published:** 2022-09-02

**Authors:** Richard Darnton, Richard Thomson, Judy McKimm

**Affiliations:** Department of Public Health and Primary Care, University of Cambridge, Cambridge.; Faculty and Student Development, School of Medical Education, Newcastle University, Newcastle.; Swansea University Medical School, Swansea University, Swansea.

Newly published research by Greenhalgh *et al* ^1^ uncovers reasons why GPs rarely do video consultations. They found that when face-to-face consultations are an option, video consultations are perceived as providing insufficient added benefit over telephone to justify their additional operational complexity. While discussing their findings, Greenhalgh *et al* reference Rogers^2^ in identifying that the adoption of any innovation is contingent on whether potential adopters perceive any benefit over existing practice. So, it appears that this innovation has not caught-on because GPs do not perceive it adds sufficient value in terms of clinical care.

At first sight this could suggest that video consulting in primary care was a temporary response to the COVID-19 pandemic and therefore has had its day. However, there is another perspective to consider — the education perspective. GPs may not perceive that video consultations confer sufficient advantage for clinical care, but what about for clinical education? From this perspective, video consultations could be a gamechanger.

## PRACTICAL BENEFITS FOR CLINICAL EDUCATION

Recent studies have elicited the perspective of primary care-based educators and medical students in relation to remote consulting.^3^^,^^4^ They have also established the feasibility of medical students undertaking consultations from their own homes.^4^ These studies suggest video consultations may confer a number of practical benefits for clinical education. First, they do not require clinically equipped consulting rooms, or for students to travel to a healthcare facility to undertake consultations. Second, they provide insight into people’s living conditions, giving valuable learning around the social context of patient presentations. They also enable clinical learners to access patients who may be too medically vulnerable to justify a face-to-face conversation where there is no discernible clinical benefit for the patient. Furthermore, they allow multiple clinical learners to participate in real-time consultations while being geographically dispersed.

Healthcare facilities are overflowing with medical students and other work-based learners. With the increasing need to accommodate new advanced practitioner roles, practices are running out of consulting rooms,^5^ potentially precipitating their withdrawal from teaching. Transporting learners to distant primary care centres may provide access to unused teaching capacity and diverse population groups, but that has a financial and environmental cost. Some use of students undertaking off-site video consultations may help mitigate these problems. While, from a situated learning point of view,^6^ this may not provide students with the same experience as being physically located at a GP practice, its potential to enable high-quality clinical supervision could partially compensate for lack of physical presence.

Video consultations could therefore offer access to educationally valuable cultural contexts/patients, unlock extra teaching capacity, and contribute to environmentally sustainable medical education.

## BENEFITS FOR THE LEARNING PROCESS

Video feedback on in-person consultations has long been a pillar of primary care education. This has, however, often been plagued by practical and technical challenges, such as inadequate audio or clunky consent processes. Video consulting, on the other hand, can circumvent many of these challenges, enabling simple production of high-quality recordings and the potential for integrated consent processes.

Furthermore, we argue that video consultation has unique affordances^7^ for clinical learning, allowing the educator to deftly guide the clinical and teaching dialogues in the ‘trialogic’ relationship between patient, learner, and clinical teacher.^8^ Whereas the in-person trialogue demands careful eye contact and proxemics to avoid the patient interacting primarily with the doctor, video allows the doctor to literally fade out, such that the learner occupies the driver’s seat, then fade back in. At this point, the GP educator can take opportunities for clarification and safety netting, for authentic involvement of the patient in the teaching encounter and for immediate feedback to the learner based on direct observation.

## IS IT ‘AUTHENTIC’ GENERAL PRACTICE TEACHING?

Quantity of exposure to authentic general practice teaching is thought to increase its attractiveness as a career choice to students,^9^ and authenticity of experience is thought to be a key ingredient.^10^ So, if GPs rarely use video consultations would we be straying beyond authentic general practice experience if students started getting primary care consulting experience this way? We believe that, as in the case of face-to-face consultations and telephone consultations, it all depends on how these consultations are delivered and supervised.

Authenticity seems to involve students *‘seeing their own patients and running their own clinics’*,^11^ and experiencing a degree of supervised clinical autonomy (rather than simply having group teaching on a patient who has been specially invited to the surgery for this purpose).^10^ Similarly, medical students undertaking remote GP consultations find the educational value and enjoyment of the experience to vary hugely depending on the degree to which they experience clinical responsibility/autonomy^3^ and consult with acutely presenting patients.^4^

So, if student video consultations involved masses of students passively observing a live video consultation, it would be no more authentic than joining a 19th century audience at the operating theatre. But, if a student is working through their own video clinic list with a tutor popping in and out of the virtual consulting room (or staying in with the camera off) then surely this is authentic? One might argue that it even offers advantages, both ergonomic and subtly pedagogic, over a conventional parallel surgery. Of course, traditional physical examination skills cannot be practised, but there is opportunity to focus closely on a gestalt assessment of wellness, and to consider what can be examined with a webcam.

## HOW MIGHT WE PROMOTE AND EMBED THESE CHANGES?

Until video consultation becomes part of our routine clinical practice, leveraging its potential for clinical education will be a challenge. However, education can be a driver for change — and rightly so given that healthcare systems (and thus patients) are dependent on the supply of trained healthcare workers.

Various approaches could be taken to promote the use of video consultations in clinical education. Quick win pilot projects (for example, GP trainees accessing specialist clinics for training) could help demonstrate the added value of video consulting in clinical education. In medical student teaching, ‘video first’ surgeries for medical student teaching could be encouraged.^3^ Clinical supervisors could be provided with effective tools, guidance, and techniques for embedding video consulting into the learning process. IT infrastructure that explicitly supports off-site medical student video surgeries might help overcome perceived barriers, as could educator development that focuses on *‘immunity to change’* ^12^ mindsets. Finally, the ability to appropriately select and use a range of consultation modalities, including video, could be added to the General Medical Council’s generic professional capabilities framework.

## CONCLUSION

Video consultations may not be the silver bullet we hoped for with regards to clinical care but we believe they could be a gamechanger in clinical education — and ultimately this benefits both learners and patients. As those involved in delivering and leading clinical education, we perceive some major educational benefits of video consulting. A growing body of evidence supports this view, but without proactive leadership to overcome potential resistance, and without wider adoption, the lessons and skills learned during the COVID-19 pandemic are at risk of being quickly forgotten. Strategies are therefore needed to enable the use of video consultations in the primary care workplace and harness the benefits they offer for clinical education.
